# P-1469. RSV-Related Knowledge, Attitudes, and Practices Among US Adults During the 2024–2025 RSV Season

**DOI:** 10.1093/ofid/ofaf695.1655

**Published:** 2026-01-11

**Authors:** Elizabeth M La, Kyli Gallington, David Singer, Zaneta Balantac, Noha S Eltoukhy, Yipin Han, Donald M Bushnell

**Affiliations:** GSK, Philadelphia, PA; Evidera, Wilmington, North Carolina; GSK, Philadelphia, PA; Evidera, Wilmington, North Carolina; GSK, Philadelphia, PA; Evidera, Wilmington, North Carolina; Evidera, Wilmington, North Carolina

## Abstract

**Background:**

Respiratory syncytial virus (RSV) vaccines have been approved for use in adults aged ≥ 60 years in the United States (US) since May 2023. During the 2024–2025 RSV season, RSV vaccination was recommended by the Advisory Committee on Immunization Practices (ACIP) for all adults ≥ 75 years of age (YoA) and adults 60–74 YoA who are at increased risk for severe RSV disease. This study assessed US adults’ RSV-related knowledge, attitudes, and practices.

**Methods:**

A cross-sectional online survey of US adults was conducted to better understand knowledge of adult RSV disease and vaccination, RSV-related attitudes, and RSV vaccination practices. The survey was fielded December 2024–January 2025 and targeted a sample size of 350 adults ≥ 75 YoA, 350 adults 60–74 YoA, 200 adults 50–59 YoA, and 200 adults 18–49 YoA. Adults 18–59 YoA were required to have ≥ 1 comorbidity that is a risk factor for severe RSV disease; the sample of adults ≥ 60 YoA targeted a mix with and without comorbidities. Adults were excluded if they were healthcare professionals (HCPs) or currently pregnant. Survey responses were analyzed descriptively.

**Results:**

The final sample included 1,227 adults (≥ 75 YoA: n=403; 60–74 YoA: n=424; 50–59 YoA: n=201; 18–49 YoA: n=199). Among all respondents, 75.3% (n=924/1,227) reported that they had heard of RSV. Most respondents (66.2%; n=811/1,225) felt that they were knowledgeable about respiratory illnesses. However, only 34.8% of respondents who had heard of RSV (n=322/924) felt that they were knowledgeable about RSV. Two-thirds of RSV-aware respondents (66.7%; n=615/922) knew that RSV vaccines were approved by the Food and Drug Administration for use in adults. RSV disease and vaccination knowledge gaps were identified (e.g., related to RSV symptoms, risk factors, and ACIP vaccine recommendations). Among respondents ≥ 60 YoA, 49.0% (n=404/825) had received an HCP recommendation for RSV vaccination and 35.3% (n=292/827) had received an RSV vaccination.

**Conclusion:**

Findings related to potential RSV knowledge and practice gaps can help inform HCP approaches to discussing RSV disease and vaccination with eligible patients and broader educational initiatives to support RSV vaccination where appropriate.

Funding: GSK (223322 [HE-RSV-012 BOD])

**Disclosures:**

Elizabeth M. La, PhD, GSK: Employed by GSK|GSK: Stocks/Bonds (Public Company) Kyli Gallington, MPH, Evidera: Employed by Evidera|GSK: Employed by Evidera, which received funding from GSK to conduct this study David Singer, PharmD, MS, GSK: Employed by GSK|GSK: Stocks/Bonds (Public Company) Zaneta Balantac, ScB, Evidera: Employed by Evidera at the time of this study|GSK: Formerly employed by Evidera, which received funding from GSK to conduct this study Noha S. Eltoukhy, PharmD, MPH, GSK: Employed by GSK|GSK: Stocks/Bonds (Public Company) Yipin Han, MHS, Evidera: Employed by Evidera|GSK: Employed by Evidera, which received funding from GSK to conduct this study Donald M. Bushnell, MA, Evidera: Employed by Evidera|GSK: Employed by Evidera, which received funding from GSK to conduct this study

